# eHealth literacy mediates the relationship between health literacy and concussion attitudes among parents in South Korea

**DOI:** 10.3389/fpubh.2026.1883113

**Published:** 2026-08-14

**Authors:** Hang A. Park, Ki Ok Ahn, Ju Ok Park, Sola Kim

**Affiliations:** 1 Department of Emergency Medicine, Hallym University, Dongtan Sacred Heart Hospital, Gyeonggi-do, Republic of Korea; 2 Department of Emergency Medicine, Myongji Hospital and Hanyang University College of Medicine, Goyang-si, Republic of Korea

**Keywords:** concussion, concussion attitude, concussion knowledge, digital health literacy, eHealth literacy, health literacy, mediation analysis

## Abstract

**Background:**

Digital technologies have become major sources of health information; however, individuals’ ability to integrate such information varies according to their health literacy (HL) and eHealth literacy (e-HL). In concussion management, accurate knowledge and appropriate attitudes are important for symptom recognition and healthcare-seeking behaviors. Nevertheless, the relationships among HL, e-HL, and concussion-related knowledge and attitudes remain poorly understood.

**Objective:**

To evaluate the associations of HL and e-HL with concussion-related knowledge and attitudes among parents in South Korea and to examine the indirect associations between HL and these outcomes through e-HL.

**Methods:**

A cross-sectional online survey was conducted among parents in South Korea. HL and e-HL were assessed using the Korean version of the HLS-EU-Q16 and the eHealth Literacy Scale (eHEALS), respectively. Outcome variables were concussion-related knowledge and attitudes. Observed-variable mediation analyses were used to statistically decompose the associations of HL with these outcomes into direct and indirect components through e-HL. An exploratory sequential mediation model additionally evaluated whether concussion-related knowledge was involved in the indirect association between HL and concussion-related attitudes.

**Results:**

In total, 260 participants completed the survey. HL was positively correlated with e-HL (*ρ* = 0.45, *p* < 0.001) and concussion-related attitudes (*ρ* = 0.19, *p* = 0.003), but not with concussion-related knowledge (ρ = 0.03, *p* = 0.657). For concussion-related knowledge, neither HL nor e-HL was significantly associated with the outcome, and no significant indirect association was observed. For concussion-related attitudes, a significant indirect association between HL and attitudes through e-HL was observed (*B* = 0.117, 95% CI: 0.022–0.220; *p* = 0.021). The exploratory sequential mediation analysis showed no significant indirect association involving concussion-related knowledge.

**Conclusion:**

Higher HL was associated with more favorable concussion-related attitudes, but not with concussion-related knowledge. An indirect association between HL and concussion-related attitudes was observed through e-HL, but not through concussion-related knowledge. This indirect association was consistent with the hypothesized model but did not establish temporal precedence or causal mediation. These findings suggest that future concussion education strategies may benefit from strengthening both HL and e-HL alongside the provision of factual concussion information.

## Introduction

Traumatic brain injury (TBI) is a major global public health concern, with crude incidence rates ranging from 47.3 to 694 per 100,000 individuals per year in European countries across all age groups and TBI severities (1). In South Korea, the age- and sex-standardized TBI incidence in 2022 was 58.8 per 100,000 people (2). In the United States (US), an estimated 1.7 million TBIs occurred annually from 2002 to 2006, resulting in 52,000 deaths each year (3). Mild TBI, commonly referred to as concussion (4), accounts for approximately 75–85% of all TBI cases and may result in persistent physical, cognitive, and emotional symptoms in individuals with delayed recovery (5). Concussions are common and are often managed outside specialized neurological care; therefore, public awareness and appropriate self-management are important public health considerations.

Health literacy (HL) refers to an individual’s ability to find, access, contextualize, and understand information needed for healthcare-related decisions (6). In concussion management, appropriate knowledge and attitudes may support symptom recognition, timely healthcare seeking, and adherence to recommended return-to-activity protocols (7). Parents play a central role in concussion prevention, recognition, and management, and effective parent education has been identified as an important component of improving concussion care and informed decision making (8). Individuals with limited HL may experience greater difficulty understanding concussion-related information and recommended recovery strategies, potentially limiting informed decision-making during concussion management. Previous study has also demonstrated associations between HL and injury-related knowledge and attitudes (9).

eHealth literacy (e-HL) extends the concept of HL to digital environments and describes an individual’s ability to seek, evaluate, and apply health information obtained from digital sources (10). As digital media and online platforms are increasingly used for health information, individuals are required to evaluate the relevance and credibility of such information and utilize it appropriately, rather than relying directly on medical professionals (10, 11). Social Cognitive Theory proposes that personal factors, behaviors, and environmental influences interact to shape health-related decision-making, with self-efficacy as a core concept (12). In digital health contexts, higher e-HL has been associated with stronger self-efficacy, which refers to confidence in one’s ability to perform a specific task (13, 14). Although e-HL is not equivalent to self-efficacy, a high level of perceived competence in accessing, evaluating, and applying digital health information may contribute to more favorable concussion-related attitudes. This provides a theoretical basis for the hypothesis that e-HL plays an indirect role in the relationship between HL and attitudes.

Despite growing evidence regarding HL and e-HL, few studies have examined their associations with concussion-related knowledge and attitudes among parents, who are key stakeholders in concussion recognition and management (8). Moreover, it remains unclear whether e-HL contributes to the associations between general HL and concussion-related knowledge and attitudes. Understanding these relationships may help inform digital health education strategies for parents. Therefore, this study examined the associations of HL and e-HL with concussion-related knowledge and attitudes among parents of school-aged children in South Korea and assessed the indirect associations between HL and these outcomes through e-HL.

## Methods

### Study design and participants

This was a cross-sectional study conducted using an online self-administered questionnaire in South Korea between January and February 2023. The present study represents a secondary analysis of a previously published survey dataset of Korean parents (15). The previous study examined concussion-related knowledge and attitudes and their associated demographic factors, whereas the present analysis examined the associations among HL, e-HL, concussion-related knowledge, and concussion-related attitudes using mediation analysis.

Participants were recruited through a research firm (GRI Research) that has supported public health research in South Korea for several decades and maintains a nationwide panel of verified members. Panel members undergo identity verification at registration, participant information is periodically updated, and multiple quality-control procedures are implemented to enhance data quality. Eligible participants were adults who were parents of at least one school-aged child aged 6–18 years. This age range was selected to include elementary, middle, and high school students, representing the target population for concussion education and parental health decision-making. Either parent was eligible to participate, and information on whether the respondent was the primary caregiver was not collected.

No additional exclusion criteria were applied. A stratified quota sampling strategy was used to achieve balanced representation across predefined child age groups. For participants with more than one eligible child, quota assignment was based on the age group of the first child (6–9, 10–12, 13–15, and 16–18 years). Recruitment continued within each stratum until the target sample size for that age group was reached. Recruitment was conducted primarily in Seoul and the surrounding metropolitan areas (Gyeonggi and Incheon). Panel members who had previously agreed to participate in online surveys and met the eligibility criteria received electronic invitations to participate. The invitation included only the study purpose, whereas detailed study information was provided during the consent process to minimize self-selection bias. Upon completion of the survey, participants were reimbursed with “reward points” equivalent to 10,000 KRW (approximately 8 USD).

### Data collection and variables

The online survey questionnaire was developed by the research team using previously validated instruments (Supplementary File 1). The translated questionnaires were reviewed by the authors and subsequently pilot-tested to assess item clarity, comprehensibility, and the feasibility of online administration. Feedback from the pilot test was incorporated into the final questionnaire before the survey was launched. The URL of the final questionnaire was integrated into the online survey platform for participant access.

The primary exposure variable was HL, which was assessed using the Korean version of the HLS-EU-Q16, a 16-item shortened form of the HLS-EU-Q47 developed by the World Health Organization Regional Office for Europe (16, 17) (Supplementary File 2). The Korean version of the HLS-EU-Q16 has been translated and validated in Korean adults, demonstrating acceptable internal consistency (Cronbach’s *α* = 0.861) (18). The questionnaire assessed three domains: healthcare (seven items), disease prevention (five items), and health promotion (four items). Responses were measured using a four-point Likert scale (“very easy,” “easy,” “difficult,” and “very difficult”). Following standard scoring procedures, responses of “easy” and “very easy” were coded as 1, whereas “difficult” and “very difficult” were coded as 0. The total HLS-EU-Q16 score ranged from 0 to 16 and was categorized as inadequate (0–8), indicating limited HL; problematic (9–12), indicating sufficient HL for some but not all health-related tasks; or adequate (13–16), indicating sufficient HL to manage most health-related tasks (19). All participants completed all 16 items, and the scale demonstrated high internal consistency (Cronbach’s *α* = 0.92).

The e-HL was assessed using the eHealth Literacy Scale (eHEALS), originally developed by Norman and Skinner (10, 20) (Supplementary File 3). The scale consists of eight items rated on a five-point Likert scale ranging from 1 (“strongly disagree”) to 5 (“strongly agree”). Higher mean scores indicate higher perceived e-HL, reflecting individuals’ self-perceived competencies in finding, evaluating, and applying online health information. In the present study, the scale demonstrated high internal consistency (Cronbach’s *α* = 0.86).

Demographic information, including sex, age, number of children, past concussion history, occupation, educational level, and monthly household income, was also collected. Monthly household income was self-reported by selecting one of eight predefined income categories (≤2 million KRW, 2.01–3 million KRW, 3.01–4 million KRW, 4.01–5 million KRW, 5.01–6 million KRW, 6.01–7 million KRW, 7.01–8 million KRW, or ≥8 million KRW). For the adjusted analyses, household income was categorized as below or above the 2022 national median monthly household income according to household size. Participants were additionally asked about their use of the internet to obtain health-related information, and the perceived usefulness of internet-based health information for health-related decision-making.

### Outcomes

The outcome variables of this study were concussion-related knowledge and attitudes, which were assessed using Korean-translated items adapted from previously developed and evaluated concussion knowledge and attitude questionnaires (9, 21) (Supplementary File 4). Concussion-related knowledge was evaluated using 30 true-or-false items covering concussion symptoms, general concepts, and the consequences of returning to activity too soon or sustaining multiple concussions. Each correct response was assigned one point, resulting in a composite knowledge score ranging from 0 to 30, with higher scores indicating greater concussion-related knowledge (21). The internal consistency of the knowledge scale was modest (Cronbach’s *α* = 0.63).

Concussion-related attitudes were assessed using seven items rated on a 7-point Likert scale ranging from 1 (“strongly disagree”) to 7 (“strongly agree”). The items assessed participants’ attitudes toward concussion symptom disclosure, reporting, awareness, and appropriate concussion management. The total attitude score ranged from 7 to 49, with higher scores indicating more favorable concussion-related attitudes toward symptom recognition, reporting and management. The attitude scale demonstrated acceptable internal consistency (Cronbach’s *α* = 0.87).

### Statistical analysis

The minimum required sample size was estimated using G*Power 3.1 with a significance level of 0.05, a power of 0.95, and a medium effect size (0.5) (22). Because no previous studies had investigated the mediation relationships among HL, e-HL, and concussion-related knowledge and attitudes, the expected mediation effect size could not be estimated *a priori*. Therefore, a medium effect size was selected according to Cohen’s conventional effect size recommendations in the absence of prior empirical estimates (23). The adequacy of the achieved sample size for the primary mediation analysis was additionally evaluated using a Monte Carlo simulation. The simulation indicated that approximately 180 participants would be required to achieve 80% power, indicating that the achieved sample size of 260 was adequate for the planned mediation analysis.

Descriptive statistics are presented as frequencies and percentages for categorical variables and as medians and interquartile ranges (IQRs) for continuous variables. Differences across HL groups were assessed using Pearson’s chi-square test or Fisher’s exact test with Monte Carlo simulation, as appropriate, for categorical variables and the Kruskal–Wallis test for continuous variables. Standardized effect sizes were reported as Cramér’s *V* for categorical variables and eta-squared based on the Kruskal–Wallis *H* statistic (*η*^2^*H*) for continuous variables. The normality of continuous variables was assessed using the Shapiro–Wilk test. Because HL, e-HL, concussion-related knowledge, and attitude scores all deviated significantly from a normal distribution (all *p* < 0.05), correlations among these variables were assessed using Spearman’s rank correlation coefficients.

**Figure 1:**
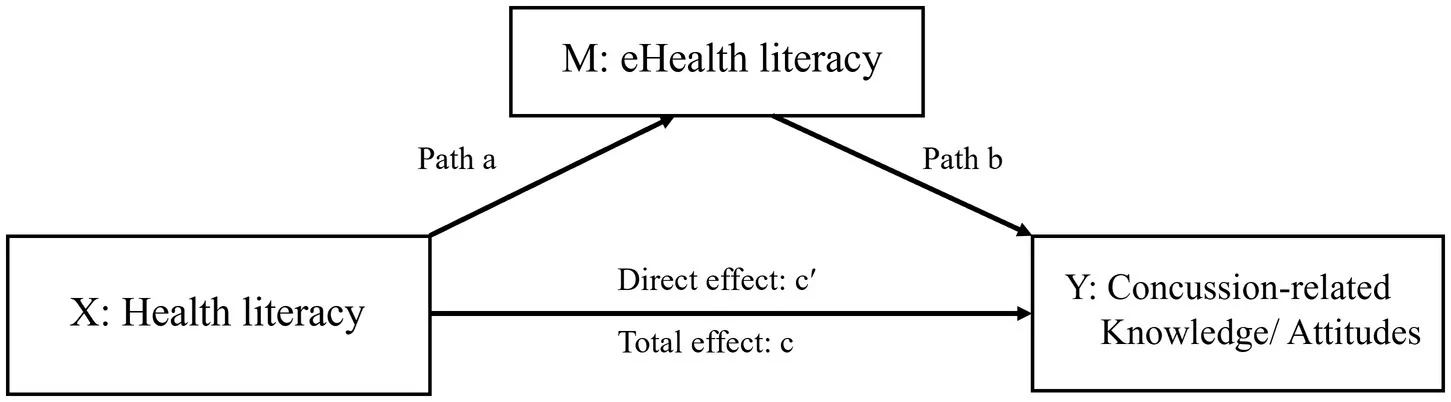
Mediation model of the association between health literacy and concussion-related attitudes through eHealth literacy.

Mediation analysis was performed as an observed-variable path analysis within a structural equation modeling framework using the *lavaan* package in R. The analyses used observed composite scores for HL, e-HL, concussion-related knowledge, and concussion-related attitudes to assess the indirect associations between HL and concussion-related knowledge and attitudes via e-HL (Figure 1). As the specified path models were just-identified (degrees of freedom = 0), conventional structural equation model fit indices were not interpreted. The models were adjusted for age, sex, parental concussion history, child concussion history, number of children, child participation in sports, educational level, below-median household income in the past year, healthcare-related occupation, and experience of internet use for health information. Covariates were selected *a priori* based on their clinical relevance and previous literature identifying demographic, socioeconomic, and clinical factors associated with HL, e-HL, and concussion-related outcomes (24, 25). Multicollinearity among the predictors was assessed using variance inflation factors (VIFs), and no evidence of problematic multicollinearity was observed (all VIFs <2.3). All participants included in the mediation analyses had complete data for the study variables; therefore, no imputation or other missing-data handling procedures were required.

Direct, indirect, and total effects were estimated using maximum likelihood estimation, with bias-corrected bootstrap confidence intervals (CIs) based on 5,000 resamples, as recommended for mediation analyses (26). As a sensitivity analysis, the mediation models were re-estimated after excluding internet health-information use experience from the adjustment set to evaluate the robustness of the findings. In a separate exploratory analysis, a sequential mediation model was estimated to examine the indirect pathway from HL to concussion-related attitudes through e-HL and concussion-related knowledge. The sequential pathway was specified as HL → e-HL → knowledge → attitude while retaining direct pathways from HL and e-HL to attitudes. For the prespecified primary analyses, a two-sided *p*-value < 0.05 was considered statistically significant. No formal adjustment for multiple comparisons was applied, and the potential for inflated Type I error was considered when interpreting the findings. For the exploratory sequential mediation analysis, unstandardized estimates and bias-corrected 99% bootstrap CIs based on 5,000 resamples were reported without *p*-values; intervals excluding zero were considered statistically supported under this more conservative criterion. All analyses were conducted using R Statistical Software (version 4.5.1; 27).

## Results

In total, 260 participants were included in the study. Of these, 120 (46.2%) had inadequate HL, 58 (22.3%) had problematic HL, and 82 (31.5%) had adequate HL. Table 1 presents the demographic and digital health–related characteristics of respondents according to HL level. The median age of all participants was 48.0 years (IQR, 40.8–50.0), with no significant difference across HL groups (*p* = 0.739; *η*^2^*H* = 0.000). In contrast, digital health–related variables differed by HL level. The proportion of respondents who perceived the internet as useful for health-related decision-making increased from 45.0% in the inadequate HL group to 67.1% in the adequate HL group (*p* = 0.034; Cramér’s *V* = 0.142). Similarly, the proportion who considered internet-based health resources important increased from 45.8 to 78.0% across the same groups (*p* < 0.001; Cramér’s *V* = 0.227). Median eHEALS scores also increased across HL categories, from 25.0 (IQR, 23.0–28.0) in the inadequate HL group to 30.5 (IQR, 26.2–33.0) in the adequate HL group (*p* < 0.001; *η*^2^*H* = 0.180).

**Table 1:** Demographic characteristics of the respondents by level of health literacy.

Variable	Total	Inadequate	Problematic	Adequate	*p*-value	Effect size
	*N* = 260	*N* = 120	*N* = 58	*N* = 82		
Age (years)	48.0 (40.8–50.0)	45.0 (40.8–50.0)	49.0 (40.2–50.0)	48.0 (41.0–49.8)	0.739	0.000^a^
Sex					0.067	0.144^b^
Male	157 (60.4)	78 (65.0)	38 (65.5)	41 (50.0)		
Female	103 (39.6)	42 (35.0)	20 (34.5)	41 (50.0)		
Number of children	2.0 (1.0–2.0)	2.0 (1.0–2.0)	2.0 (1.0–2.0)	2.0 (1.0–2.0)	0.408	0.000^a^
Child participation in sports					0.108	0.131^b^
No	180 (69.2)	79 (65.8)	37 (63.8)	64 (78.0)		
Yes	80 (30.8)	41 (34.2)	21 (36.2)	18 (22.0)		
Child concussion history					0.189	0.113^b^
No	201 (77.3)	88 (73.3)	44 (75.9)	69 (84.1)		
Yes	59 (22.7)	32 (26.7)	14 (24.1)	13 (15.9)		
Parent concussion history					0.615	0.061^b^
No	214 (82.3)	96 (80.0)	48 (82.8)	70 (85.4)		
Yes	46 (17.7)	24 (20.0)	10 (17.2)	12 (14.6)		
Level of education					0.307	0.096^b^
High school graduate or below	28 (10.8)	11 (9.2)	4 (6.9)	13 (15.9)		
College graduate	195 (75.0)	90 (75.0)	44 (75.9)	61 (74.4)		
Postgraduate school	37 (14.2)	19 (15.8)	10 (17.2)	8 (9.8)		
Household income					0.461	0.077^b^
<median	177 (68.1)	86 (71.7)	39 (67.2)	52 (63.4)		
≥median	83 (31.9)	34 (28.3)	19 (32.8)	30 (36.6)		
Healthcare-related job					0.301	0.096^b^
No	241 (92.7)	113 (94.2)	55 (94.8)	73 (89.0)		
Yes	19 (7.3)	7 (5.8)	3 (5.2)	9 (11.0)		
Internet use experience for health information					0.674	0.055^b^
No	31 (11.9)	12 (10.0)	8 (13.8)	11 (13.4)		
Yes	229 (88.1)	108 (90.0)	50 (86.2)	71 (86.6)		
Perceived usefulness of the internet for health decisions					0.034	0.142^b^
Not useful	12 (4.6)	6 (5.0)	2 (3.4)	4 (4.9)		
Unsure	106 (40.8)	60 (50.0)	23 (39.7)	23 (28.0)		
Useful	142 (54.6)	54 (45.0)	33 (56.9)	55 (67.1)		
Perceived importance of the internet for health resources					<0.001	0.227^b^
Not important	8 (3.1)	8 (6.7)	0 (0.0)	0 (0.0)		
Unsure	100 (38.5)	57 (47.5)	25 (43.1)	18 (22.0)		
Important	152 (58.5)	55 (45.8)	33 (56.9)	64 (78.0)		
eHEALS	27.0 (24.0–30.2)	25.0 (23.0–28.0)	28.0 (25.0–30.0)	30.5 (26.2–33.0)	<0.001	0.180^a^

Categorical variables are presented as frequencies and percentages, and continuous variables are presented as medians and interquartile ranges. Differences across HL groups were evaluated using Pearson’s chi-square test or Fisher’s exact test with Monte Carlo simulation, as appropriate, for categorical variables and the Kruskal–Wallis test for continuous variables.

Effect sizes are reported as Cramér’s *V* for categorical variables and eta-squared based on the Kruskal–Wallis *H* statistic (*η*^2^*H*) for continuous variables. For *η*^2^*H*, values of 0.01, 0.06, and 0.14 indicate small, medium, and large effects, respectively. For Cramér’s *V*, small, medium, and large effects were defined as 0.10/√df*, 0.30/√df*, and 0.50/√df*, respectively, where df* = min (*r* − 1, *c* − 1). ^a^Eta-squared based on the Kruskal–Wallis *H* statistic (*η*^2^*H*).

^b^Cramér’s *V*. eHEALS, eHealth Literacy Scale; HL, health literacy; IQR, interquartile range.

**Figure 2:**
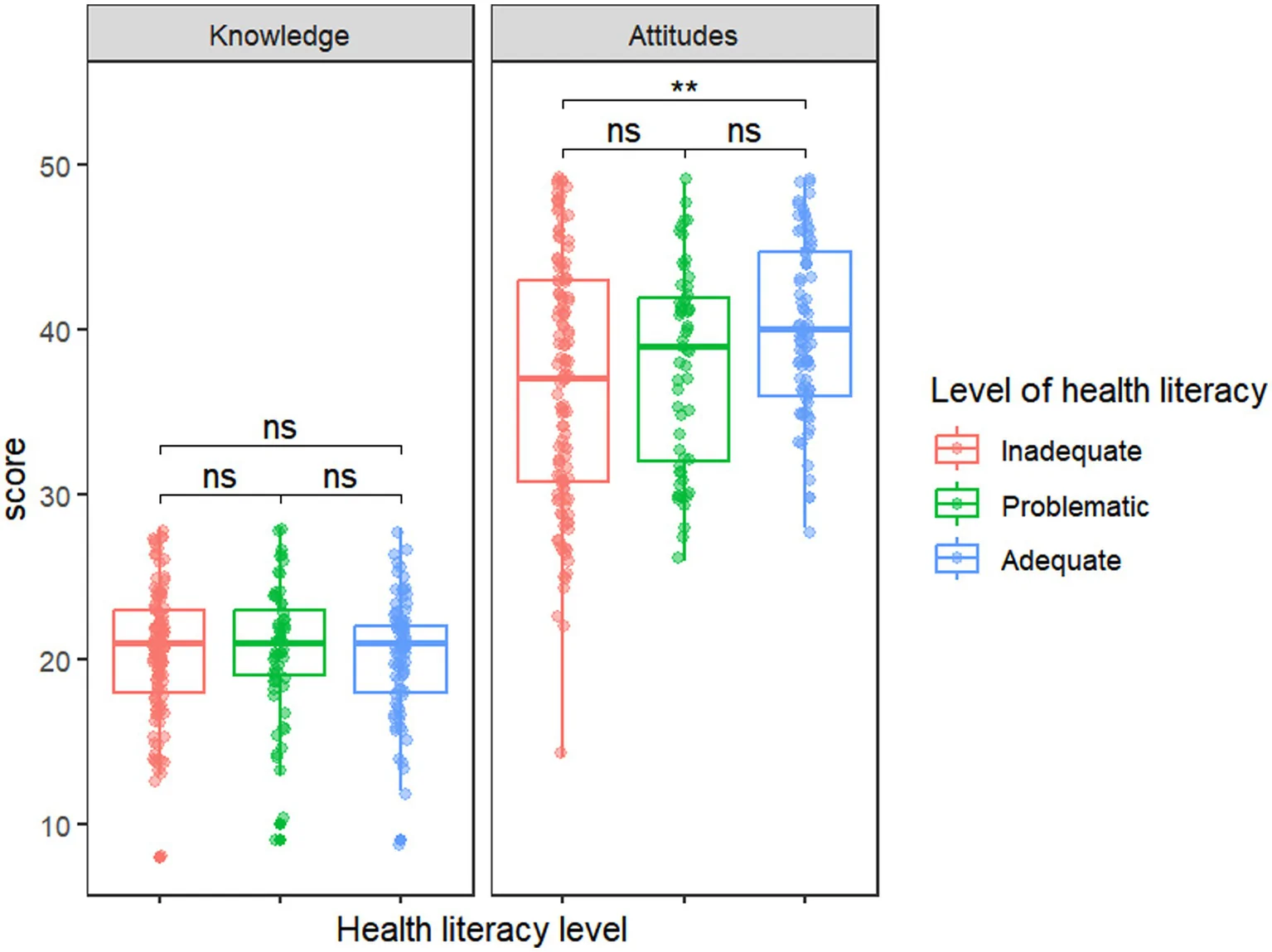
Concussion-related knowledge and Concussion-related knowledge and attitudes scores according to health literacy level. Pairwise comparisons using the Wilcoxon rank sum test were performed. ns, non-significant; *: FDR *p*-value < 0.05; **: FDR *p*-value < 0.01; ***: FDR *p*-value < 0.001.

Figure 2 shows the concussion-related knowledge and attitude scores according to HL levels. The median (IQR) knowledge scores were similar across the inadequate, problematic, and adequate HL groups at 21.0 (18.0–23.0), 21.0 (19.0–23.0), and 21.0 (18.0–22.0), respectively, with no significant difference among the groups (*p* = 0.94). In contrast, concussion-related attitude scores differed significantly across HL levels (inadequate: 37.0 [30.8–43.0]; problematic: 39.0 [32.0–42.0]; and adequate: 40.0 [36.0–44.8]) (*p* = 0.01).

**Figure 3:**
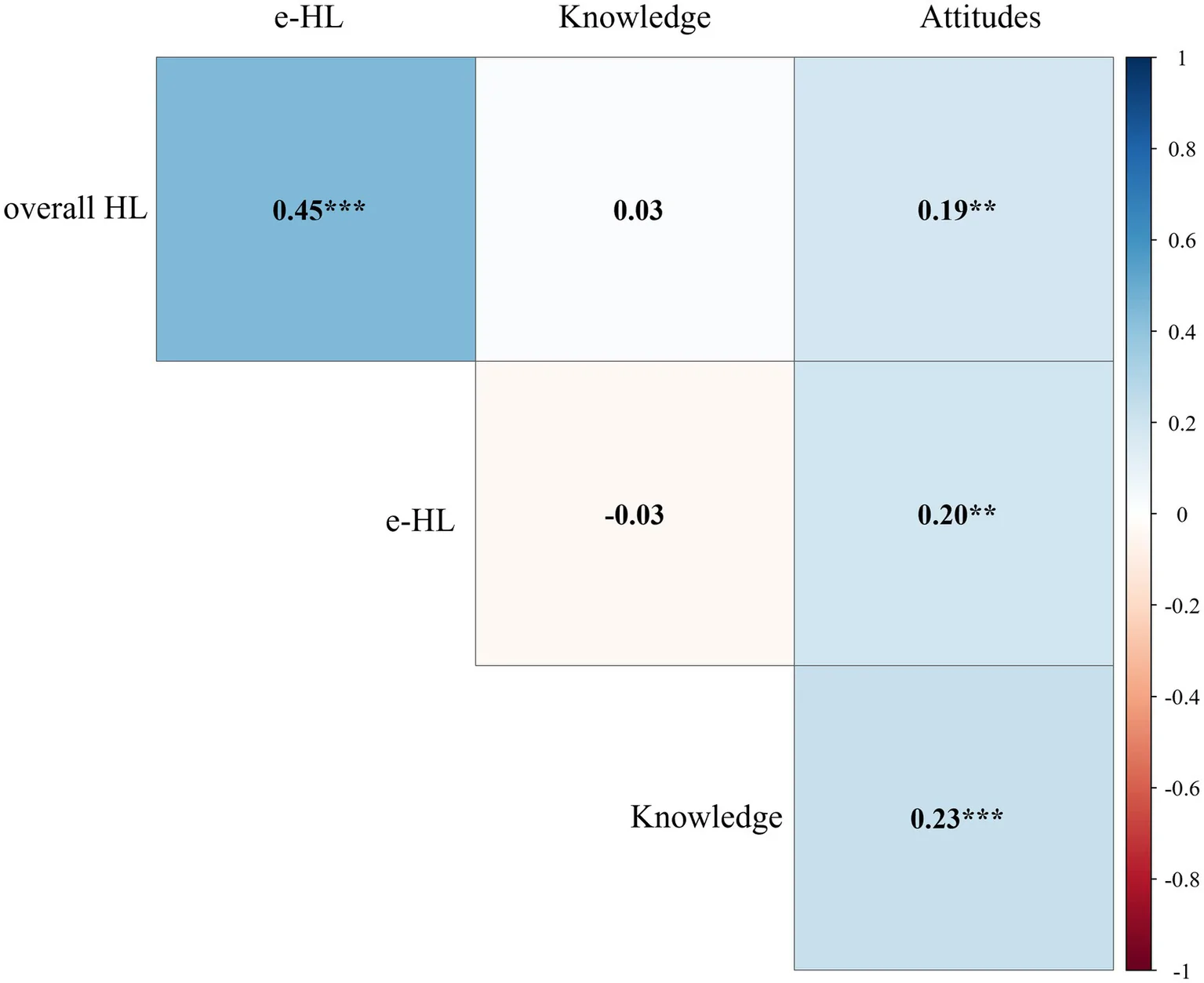
Correlations among health literacy, eHealth literacy, concussion-related knowledge, and concussion-related attitudes. HL, health literacy; e-HL, eHealth literacy. Spearman’s rank correlation coefficients (*ρ*) are presented. Analyses were based on the full analytic sample (*n* = 260). Statistical significance is indicated as *p < 0.05, **p < 0.01, and ***p < 0.001.

The correlation analysis is presented in Figure 3. HL was moderately positively correlated with e-HL (*ρ* [correlation coefficient] = 0.45, *p* < 0.001) and weakly correlated with concussion-related attitudes (*ρ* = 0.19, *p* = 0.003). However, HL was not significantly correlated with concussion-related knowledge (*ρ* = 0.03, *p* = 0.657). Similarly, e-HL was positively correlated with concussion-related attitudes (*ρ* = 0.20, *p* = 0.001) but not significantly correlated with concussion-related knowledge (*ρ* = −0.03, *p* = 0.583). Concussion-related knowledge was weakly positively correlated with concussion-related attitudes (*ρ* = 0.23, *p* < 0.001).

Table 2 presents the results of the mediation analysis examining the indirect associations of HL with concussion-related knowledge and attitudes through e-HL. HL was positively associated with e-HL in both mediation models (unstandardized coefficients [B]: 0.435; 95% CI: 0.312 to 0.564; *p* < 0.001). For the concussion-related knowledge outcome, neither e-HL (*B*: 0.012; 95% CI: −0.083 to 0.113; *p* = 0.819) nor HL (*B*: 0.002; 95% CI: −0.096 to 0.098; *p* = 0.966) was significantly associated with knowledge. The indirect effect was not significant (*B*: 0.005; 95% CI: −0.037 to 0.051; *p* = 0.822) and the total effect was also not significant (*B*: 0.007; 95% CI: −0.082 to 0.096; *p* = 0.872).

**Table 2:** Mediation analysis of eHealth literacy in the associations between health literacy, concussion-related knowledge and attitudes.

Path	Estimate (B)	95% bootstrap CI	*p*-value	*R* ^2^
Outcome: Concussion-related Knowledge (CK)				
Step I: HL → e-HL (Path a)	0.435	0.312 to 0.564	<0.001	e-HL: 0.259
Step II: e-HL → CK (Path b)	0.012	−0.083 to 0.113	0.819	
Step III: HL → CK (Direct association, c′)	0.002	−0.096 to 0.098	0.966	CK: 0.187
Indirect association (a × b)	0.005	−0.037 to 0.051	0.822	
Total association (c)	0.007	−0.082 to 0.096	0.872	
Outcome: Concussion-related Attitudes (CA)				
Step I: HL → e-HL (Path a)	0.435	0.312 to 0.564	<0.001	e-HL: 0.259
Step II: e-HL → CA (Path b)	0.269	0.052 to 0.482	0.014	
Step III: HL → CA (Direct association, c′)	0.180	−0.023 to 0.400	0.101	CA: 0.179
Indirect association (a × b)	0.117	0.022 to 0.220	0.021	
Total association (c)	0.297	0.111 to 0.487	0.002	

B, unstandardized coefficient; CA, concussion-related attitudes; CI, confidence interval; CK, concussion-related knowledge; HL, health literacy; e-HL, eHealth literacy. Mediation analyses were conducted using maximum likelihood estimation. Unstandardized estimates and bias-corrected 95% bootstrap confidence intervals based on 5,000 resamples are reported. *R*^2^ values indicate the proportion of variance explained in each endogenous variable. The model was adjusted for age, sex, parental concussion history, child concussion history, number of children, child sports participation, education level, household income, healthcare-related occupation, and internet health-information use experience. These estimates represent statistical decompositions under the hypothesized models and do not establish temporal ordering or causality.

In contrast, for the attitude outcome, e-HL was positively associated with concussion-related attitudes (*B*: 0.269; 95% CI: 0.052 to 0.482; *p* = 0.014), whereas the direct effect of HL on concussion-related attitudes was not statistically significant (*B*: 0.180; 95% CI: −0.023 to 0.400; *p* = 0.101). The indirect effect of HL on concussion-related attitudes through e-HL was statistically significant (*B*: 0.117; 95% CI: 0.022 to 0.220; *p* = 0.021), and the total effect was also significant (*B*: 0.297; 95% CI: 0.111 to 0.487; *p* = 0.002). Sensitivity analyses excluding internet health-information use experience from the adjusted models produced similar results. The indirect association between HL and concussion-related attitudes through e-HL remained statistically significant, whereas no significant indirect association was observed for concussion-related knowledge (Table 3). These findings support the robustness of the primary conclusions.

**Table 3:** Sensitivity mediation analysis excluding internet health-information use experience from the adjusted models.

Path	Estimate (B)	95% bootstrap CI	*p*-value	*R* ^2^
Outcome: Concussion Knowledge (CK)				
Step I: HL → e-HL (Path a)	0.433	0.311 to 0.560	<0.001	e-HL: 0.257
Step II: e-HL → CK (Path b)	0.023	−0.079 to 0.130	0.664	
Step III: HL → CK (Direct effect, c′)	−0.010	−0.110 to 0.088	0.834	CK: 0.134
Indirect association (a × b)	0.010	−0.035 to 0.059	0.670	
Total association (c)	0.000	−0.089 to 0.087	0.995	
Outcome: Concussion Attitudes (CA)				
Step I: HL → e-HL (Path a)	0.433	0.311 to 0.560	<0.001	e-HL: 0.257
Step II: e-HL → CA (Path b)	0.290	0.064 to 0.507	0.010	
Step III: HL → CA (Direct association, c′)	0.157	−0.040 to 0.370	0.136	CA: 0.138
Indirect association (a × b)	0.125	0.027 to 0.232	0.016	
Total association (c)	0.283	0.100 to 0.475	0.003	

B, unstandardized coefficient; CA, concussion-related attitudes; CI, confidence interval; CK, concussion-related knowledge; HL, health literacy; e-HL, eHealth literacy. Mediation analyses were conducted using maximum likelihood estimation. Unstandardized estimates and bias-corrected 95% bootstrap confidence intervals based on 5,000 resamples are reported. *R*^2^ values indicate the proportion of variance explained in each endogenous variable. The model was adjusted for age, sex, parental concussion history, child concussion history, number of children, child sports participation, education level, household income, and healthcare-related occupation.

In an exploratory analysis, a sequential mediation model specifying the pathway HL → e-HL → concussion-related knowledge → concussion-related attitudes was estimated. HL remained positively associated with e-HL, whereas neither HL nor e-HL was associated with knowledge. The sequential indirect association of HL with attitudes through e-HL and knowledge was not statistically supported (*B* = 0.002; 99% bootstrap CI: −0.028 to 0.031). In contrast, the indirect association through e-HL alone remained statistically supported under the more conservative criterion applied to the exploratory analysis (*B* = 0.115; 99% bootstrap CI: 0.004 to 0.227). The total indirect association was not statistically supported (*B* = 0.118; 99% bootstrap CI: −0.002 to 0.280), whereas the total association between HL and attitudes remained positive (*B* = 0.297; 99% bootstrap CI: 0.047 to 0.541) (Table 4). These estimated direct and indirect associations represent statistical decompositions under the hypothesized model and do not establish temporal ordering or causality.

**Table 4:** Exploratory sequential mediation analysis of the association between health literacy and concussion-related attitudes through eHealth literacy and concussion-related knowledge.

Path	Estimate (B)	99% bootstrap CI	*R* ^2^
Outcome: eHealth literacy			
HL → e-HL (Path a)	0.435	0.270 to 0.602	e-HL: 0.259
Outcome: Concussion-related Knowledge (CK)			
e-HL → CK (Path d)	0.012	−0.118 to 0.140	
HL → CK (Path c′K)	0.002	−0.127 to 0.139	CK: 0.187
Indirect association through e-HL (a × d)	0.005	−0.055 to 0.066	
Outcome: Concussion-related Attitudes (CA)			
CK → CA (Path e)	0.447	0.145 to 0.778	
e-HL → CA (Path b)	0.264	0.002 to 0.551	
HL → CA (Direct association, c′a)	0.179	−0.090 to 0.437	CA: 0.220
Specific indirect associations with concussion-related attitudes			
HL → e-HL → CA (a × b)	0.115	0.004 to 0.227	
HL → CK → CA (c′K × e)	0.001	−0.062 to 0.064	
HL → e-HL → CK → CA (a × d × e)	0.002	−0.028 to 0.031	
Total indirect association with CA	0.118	−0.002 to 0.280	
Total association with CA	0.297	0.047 to 0.541	

B, unstandardized coefficient; CA, concussion-related attitudes; CI, confidence interval; CK, concussion-related knowledge; HL, health literacy; e-HL, eHealth literacy. The sequential mediation analysis was exploratory. Unstandardized coefficients and bias-corrected bootstrap 99% confidence intervals based on 5,000 resamples are reported. Confidence intervals excluding zero indicate statistical support under the conservative inferential criterion applied to this exploratory analysis. *R*^2^ values indicate the proportion of variance explained in each endogenous variable. The model was adjusted for age, sex, parental concussion history, child concussion history, number of children, child sports participation, education level, household income, healthcare-related occupation, and internet health-information use experience.

## Discussion

This study evaluated the associations of HL and e-HL with concussion-related knowledge and attitudes among parents in South Korea. Higher HL was associated with more favorable concussion-related attitudes but not with concussion-related knowledge. Mediation analyses further identified a significant indirect association between HL and concussion-related attitudes through e-HL, whereas no indirect association was observed for concussion-related knowledge. An exploratory sequential mediation analysis additionally showed that the indirect association was observed through e-HL alone and not through concussion-related knowledge.

The central finding of this study was the divergence between the associations observed for concussion-related attitudes and factual knowledge. HL and e-HL were not associated with concussion-related knowledge, whereas the association between HL and concussion-related attitudes was primarily observed through e-HL. This suggests that literacy-related competencies may be more closely related to concussion-related attitudes than to factual concussion knowledge. Knowledge primarily reflects recognition and understanding of concussion-related facts, whereas attitudes involve evaluative judgments, perceived importance, and willingness to respond appropriately to concussion-related situations (28, 29). Previous concussion research has similarly suggested that factual knowledge may be associated with favorable attitudes or behavioral intentions but may not be sufficient to determine them, as psychosocial factors also contribute to attitudinal formation (28). Educational interventions focused primarily on increasing factual knowledge have likewise shown limited effectiveness in changing concussion-related educational outcomes (30).

Concussion-related knowledge was positively associated with attitudes in the exploratory sequential model, indicating that greater factual knowledge may still be relevant to favorable attitudes. However, knowledge did not account for the association between HL and attitudes because neither HL nor e-HL was associated with knowledge. This pattern suggests that knowledge and attitudes are related but qualitatively distinct constructs. Factual knowledge may provide a foundation for informed judgments, whereas the development of favorable attitudes may additionally require confidence, motivation, perceived control, and an ability to interpret information in relation to a specific situation. Thus, greater knowledge may support favorable attitudes without constituting the principal indirect pathway linking HL with attitudes.

One possible explanation for these findings is that e-HL encompasses competencies beyond the acquisition of factual knowledge. Individuals with higher e-HL may have greater confidence in accessing, interpreting, and applying health information (10, 11, 31). These competencies may influence how individuals assess the seriousness of a concussion, interpret recommendations, and judge the importance of reporting symptoms and ensuring appropriate management. Therefore, e-HL may be more directly related to attitude formation than to the simple recall of facts. This explanation is conceptually consistent with Social Cognitive Theory, which identifies self-efficacy as an important determinant of health-related motivation, decision-making, and behavior (12). Greater confidence in using health information, together with stronger appraisal skills and perceived control, may therefore be associated with more favorable concussion-related attitudes even when factual knowledge does not differ substantially (31, 32). Previous studies have also reported that health-related attitudes are associated not only with knowledge but also with self-efficacy, motivation, beliefs, and perceived control over health-related decisions (33).

In contrast, the absence of significant associations between HL and e-HL and concussion-related knowledge suggests that general literacy-related competencies do not necessarily translate into greater factual knowledge of a specific condition. Acquiring concussion-specific knowledge may depend on prior exposure, interest, motivation, cognitive engagement, and opportunities to receive relevant education. Consistent with this interpretation, Turner et al. reported a mediating role of need for cognition in the association between HL and knowledge of concussion symptoms among collegiate athletes, suggesting that literacy-related competencies alone may be insufficient to improve concussion-related knowledge (29). Taken together, these findings suggest that e-HL may be associated with concussion-related attitudes through processes involving critical evaluation, personalization, and application of information rather than through greater factual knowledge alone. However, because all variables were measured concurrently, the observed indirect association between HL and attitudes through e-HL should be interpreted as a statistical decomposition under the hypothesized model rather than evidence of a causal mediation pathway. Temporal ordering cannot be established, and reverse or bidirectional relationships and unmeasured confounding remain possible.

Although HL was not directly associated with concussion-related knowledge or attitudes after accounting for e-HL and covariates, it remains an important public health consideration. In this study, 46.2% of participants were classified as having inadequate HL, showing that limited HL is common. This prevalence is broadly comparable to the 50.7% reported in the 2021 Korean Health Panel Survey using the same HLS-EU-Q16 instrument (34) and the 39.6% reported in the first nationwide HL assessment in South Korea using a different HL measurement instrument (35). In addition, HL was positively associated with e-HL, suggesting that foundational HL may be closely related to parents’ perceived ability to access, evaluate, and use digital health information (36–38). Therefore, the findings do not suggest that HL is unimportant; rather, they suggest that HL alone may be insufficient to explain concussion-specific knowledge or attitudes without considering digital health competencies.

These findings should also be interpreted in the context of South Korea’s highly digitalized infrastructure and healthcare environment. Digital health information is increasingly integrated into health information seeking and healthcare use in South Korea, which may increase the relevance of e-HL to parental health decision-making. Therefore, the observed associations may not be directly generalizable to countries with less developed digital infrastructure, lower access to online health information, or different healthcare delivery systems. Cultural attitudes toward seeking health information, trust in healthcare professionals, and parental responsibility regarding health-related decisions for their children can also influence how parents interpret and apply information about concussions. Future research should investigate whether similar associations are observed across different digital, cultural, and healthcare system environments.

The present findings have several implications for concussion education and public health strategies. Educational strategies may benefit from targeting both foundational HL and e-HL rather than focusing primarily on increasing factual knowledge. Previous studies have similarly reported limited success in changing concussion-related educational outcomes through knowledge-based approaches (30, 39). As HL reflects broader abilities to obtain, understand, and apply health information beyond formal educational attainment (40, 41), interventions may need to strengthen HL directly rather than relying on educational background. Parent-focused concussion education programs may therefore benefit from complementing factual information with strategies that enhance individuals’ ability to critically evaluate and appropriately apply digital health information during health-related decision-making. Given that both HL and e-HL are potentially modifiable through targeted interventions (42–44), future educational programs may benefit from adopting an integrated approach that addresses both foundational HL and digital health competencies.

This study has several strengths, including the simultaneous evaluation of HL, e-HL, concussion-related knowledge, and concussion-related attitudes, together with mediation and exploratory sequential mediation analyses to evaluate both direct and indirect associations. Despite these strengths, the observed associations were modest, and the mediation model explained only a limited proportion of the variance in concussion-related attitudes. This is not unexpected because health-related attitudes are influenced by multiple psychological, interpersonal, social, and contextual factors beyond HL and e-HL (33). HL and e-HL should be viewed as components of a broader set of determinants associated with concussion-related attitudes.

Nevertheless, several limitations should be considered when interpreting the findings. First, the cross-sectional design precludes establishing temporal ordering and causal relationships among HL, e-HL, and concussion-related attitudes. The mediation analysis represents a statistical decomposition of the observed associations into direct and indirect components under the hypothesized model, not a test of causal mediation. Because the variables were measured concurrently, the findings depend on the specified model, and alternative directional models may fit the data equally well. Thus, the specified pathway represents one plausible model among several, and the significant indirect association through e-HL should be interpreted as being consistent with, rather than confirming, the hypothesized model. Reverse or bidirectional relationships and unmeasured confounding cannot be excluded.

Second, participants were recruited from an online research panel rather than through probability-based sampling. Therefore, the sample was not designed to be nationally representative of South Korean parents, and the findings cannot be generalized beyond populations with similar sociodemographic and digital profiles. In particular, the sample was predominantly middle-aged, included a greater proportion of fathers than mothers, and had a high proportion of college-educated respondents, which may not reflect the broader parent population. Individuals with greater digital familiarity or a stronger interest in health-related topics may also have been more likely to participate, potentially introducing selection bias. Information on response rates and the characteristics of non-respondents was unavailable; therefore, the potential for non-response bias could not be evaluated, and the direction or magnitude of any resulting selection bias could not be determined. The findings may also be less applicable to parents with limited digital access. Future research should prioritize the recruitment of more diverse and representative samples.

Third, HL, e-HL, and concussion-related outcomes were assessed using self-reported questionnaires administered within a single online survey, which may have introduced common method bias. Although the survey was anonymous, social desirability bias may have influenced responses, particularly for concussion-related attitudes. In addition, this study assessed concussion-related knowledge and attitudes rather than actual concussion management behaviors; therefore, the clinical significance of the observed associations remains uncertain. eHEALS also measures self-perceived e-HL rather than objectively assessed digital health skills, and perceived competence may differ from actual ability to critically evaluate online concussion information. Future studies should use multiple data sources, scenario-based assessments, and longitudinal or intervention designs to evaluate whether improvements in HL and e-HL lead to meaningful changes in concussion management behaviors.

Fourth, findings related to concussion knowledge should be interpreted cautiously because the knowledge scale showed limited internal consistency (Cronbach’s *α* = 0.63), below the conventionally accepted threshold of 0.70. The concussion knowledge questionnaire was designed as a performance-based assessment covering multiple domains of factual concussion knowledge rather than as a unidimensional psychometric scale (45). Accordingly, limited internal consistency was not unexpected. Although the items were adapted from previously developed instruments that had been used in athlete and parent populations (9, 21), the Korean version underwent expert review for face and content validity and pilot testing for clarity and comprehensibility but did not undergo separate formal psychometric validation. Measurement error may therefore have attenuated the observed associations and contributed to the null findings.

Finally, no formal adjustment for multiple comparisons was applied to the prespecified primary mediation models, and the possibility of Type I error cannot be excluded. The sequential mediation analysis was therefore designated as exploratory and evaluated using 99% bootstrap confidence intervals. Its findings should be considered hypothesis-generating and require confirmation in prospectively specified studies. Future research should use more reliable and extensively validated knowledge measures and longitudinal or intervention designs to establish temporal ordering and determine whether HL and e-HL are associated with meaningful changes in concussion management behaviors.

## Conclusion

Higher HL was associated with more favorable concussion-related attitudes, whereas neither HL nor e-HL was significantly associated with concussion-related knowledge. A significant indirect association between HL and concussion-related attitudes was observed through e-HL but not through concussion-related knowledge. These findings suggest that future concussion education and public health strategies may benefit from strengthening both HL and e-HL alongside the provision of factual concussion information.

## Data Availability

The raw data supporting the conclusions of this article will be made available by the authors, without undue reservation.
