# Supplementary material for: eHealth literacy mediates the relationship between health literacy and concussion attitudes among parents in South Korea
Source: Front Public Health. 2026 Aug 14;14:1883113. doi: 10.3389/fpubh.2026.1883113 (PMC13522205; doi:10.3389/fpubh.2026.1883113)
Supplement: Supplementary file 1 [file Supplementary_file_1.docx]

Survey on knowledge and attitudes toward concussion

Thank you for participating in our survey, which is part of the National Research Foundation of Korea’s study on “Development and application of an educational program for management of mild traumatic brain injury based on a non-face-to-face education platform,” and seeks to determine health information literacy and knowledge and attitudes about concussion among the general public.

The survey will take approximately 20-30 minutes to complete. Those who complete the survey will receive a credit worth 10,000 won. Your answers will be kept completely confidential, will not be used individually for any purpose, and will only be used for academic statistical analysis.

**Concussion**

A concussion is a temporary abnormality in brain function that occurs when an external physical shock is applied to the head but is not accompanied by structural damage. It is also referred to as mild brain injury. The following questionnaire covers the symptoms of concussion. Please, select “Yes” if you think this is something that can happen or a symptom a person may feel after a concussion, or “No” if not.

| Questions for symptoms or sign of concussion | Yes | No |
| --- | --- | --- |
| 1. Things smell funny |  |  |
| 2. Things taste funny |  |  |
| 3. Problems remembering things |  |  |
| 4. Stiffness of joints |  |  |
| 5. Fuzzy vision |  |  |
| 6. Black eye |  |  |
| 7. Bleeding from the ear |  |  |
| 8. Bleeding from the mouth |  |  |
| 9. Bleeding from the nose |  |  |
| 10. Confusion |  |  |
| 11. Fever |  |  |
| 12. Dizziness |  |  |
| 13. Loss of Consciousness |  |  |
| 14. Nausea |  |  |
| 15. Numbness in the arms |  |  |
| 16. Skin rash |  |  |
| 17. Weakness in neck movement |  |  |
| 18. Chest pain |  |  |
| 19. Trouble understanding things |  |  |

**The survey examines general knowledge related to concussions. Choose the answer to the question that you think is most appropriate.**

1. Concussion only occurs when consciousness is lost (1) Yes (2) No

2. Concussion is an injury to which of the following areas? (Choose just one)

(1) Skull (2) Face (3) Brain (4) Neck (5) I don’t know which part of the concussion is damaged.

3. Select all that you believe occur in people who return to physical activity too soon after a concussion.

□ Nothing happens

□ Increases the chance of having another concussion

□ Skin rash occurs

□ Brain damage occurs

□ I’m not sure what will happen

4. Select all that you believe occur in people who have suffered multiple concussions

□ Nothing happens

□ Increases the chance of having another concussion

□ Skin rash occurs

□ Brain damage occurs

□ Difficulty remembering

□ I’m not sure what will happen

**This survey asks about your attitudes toward concussions. Please rate the following items on a 7-point scale.**

| Seriousness | Never - Extremely | | | | | | |
| --- | --- | --- | --- | --- | --- | --- | --- |
| How serious do you think it is when a person experiences a headache and dizziness after a blow to the head or body? |  |  |  |  |  |  |  |
| How important do you think it is for a person not to participate in physical activity when experiencing signs and symptoms of a concussion? |  |  |  |  |  |  |  |
| How important do you think it is to be informed about how concussions happen? |  |  |  |  |  |  |  |
| How important do you think it is to be informed about how concussions con be prevented? |  |  |  |  |  |  |  |
| How important do you think it is to know the steps to follow if a person has a concussion? |  |  |  |  |  |  |  |
| How important is it for a person to report possible concussion symptoms to a medical professional or caregiver? |  |  |  |  |  |  |  |
| Generally, education about concussion is not sufficient. |  |  |  |  |  |  |  |

**The following questions are related to concussions you have experienced in your lifetime.**

1. Have you ever experienced a concussion?
(1) No (2) Yes
2. Have you ever been to the hospital due to a concussion?
(1) No (2) Yes
3. Have you ever visited a hospital as a guardian of a concussion patient?
(1) No (2) Yes
4. Have you ever received education related to concussions?
(1) No (2) Yes

**Below is a survey about your ability to understand health-related information. For each of the following items, please indicate how difficult you find it, using a scale of 1 to 4, with 1 being “very difficult” and 4 being “very easy.**

| Items | Very easy | Easy | Difficult | Very difficult |
| --- | --- | --- | --- | --- |
| Find information on treatments of illness that concern you |  |  |  |  |
| Find out where to get professional help when you are ill? |  |  |  |  |
| Understand what your doctor says to you? |  |  |  |  |
| Understand your doctor’s or pharmacist’s instruction on how to take a prescribed medicine |  |  |  |  |
| Judge when you may need to get a second opinion from another doctor? |  |  |  |  |
| Use information the doctor gives you to make decisions about your illness? |  |  |  |  |
| Follow instructions from your doctor or pharmacist? |  |  |  |  |
| Find information on how to manage mental health problems like stress or depression? |  |  |  |  |
| Understand health warnings about behavior such as smoking, low physical activity and drinking too much? |  |  |  |  |
| Understand why you need health screenings? |  |  |  |  |
| Judge if the information on health risks in the media is reliable? |  |  |  |  |
| Decide how you can protect yourself from illness based on information in the media? |  |  |  |  |
| Find out about activities that are good for your mental well-being? |  |  |  |  |
| Understand advice on health from family members or friends? |  |  |  |  |
| Understand information in the media on how to get healthier? |  |  |  |  |
| Judge which everyday behavior is related to your health? |  |  |  |  |

**The following questions ask about your opinions and experiences using the internet for health-related purposes.**

1. Have you ever used the Internet to search for health information??

□ No

□ Yes

2. How useful do you feel the internet is in helping you in making decisions about your health?

□ Not useful at all

□ Not useful

□ Unsure

□ Useful

□ Very useful

3. How important is it for you to be able to access health resources on the internet?

□ Not important at all

□ Not important

□ Unsure

□ Important

□ Very important

**This survey covers your demographic characteristics and will not be used for any purpose other than academic statistical analysis.**

1. How old are you?

2. What is your gender? (1) Male (2) Female

3. What is your marital status?

(1) Single (2) Married/common-law marriage (3) Separated/widowed/divorced

4. How many family members do you live with?

(1) None (2) 1 person (3) 2 people (4) 3 or more

5. Do you have any sports-related experience or coaching experience?

(1) None (2) Yes

6. Do you have experience working in the health and medical field (doctor, nurse, dentist, midwife, oriental medicine doctor, physical therapist, occupational therapist, radiologist, clinical pathologist, plaque hygienist and dental technician, pharmacist, nursing assistant, emergency rescue technician, etc.)?

(1) No (2) Yes

7. What is your highest educational level?

(1) Below high school graduate

(2) High school graduate

(3) College student or graduate

(4) Graduate school graduate or higher

8. Which of the following best describes your occupation?

(1) Professional/free worker (e.g. doctor, lawyer, university professor, artist, religious figure, journalist, high-income freelancer)

(2) Office/technical worker (e.g. office worker at general companies, technical worker, elementary, middle, and high school teacher, web designer, computer programmer, etc.)

(3) Skilled worker (e.g. heavy equipment/truck driver, electronics/home appliance technician, lathes/woodworker, skilled worker, etc.)

(4) Self-employment (owners of small businesses with 9 or fewer employees, family workers, private taxi drivers, pharmacists, etc.)

(5) Management/administrative position (e.g., level 5 or higher civil servant, principal, corporate manager or higher)

(6) Agriculture, fishing, forestry, livestock farming, and horticulture (including family workers)

(7) Sales/service position (e.g. store clerk, salesman, insurance planner, etc.)

(8) General work (e.g. civil engineering field work, cleaning, janitor, etc.)

(9) Student

(10) Other (please specify) (________________________________)

9. What is your average monthly household income (not personal income)?

(1) Below 2 million won (2) 2.01~3 million won (3) 3.01~4 million won

(4) 4.01~5 million won (5) 5.01~6 million won (6) 6.01~7 million won

(7) 7.01~8 million won (8) 8 million won or more
