# Supplementary material for: eHealth literacy mediates the relationship between health literacy and concussion attitudes among parents in South Korea
Source: Front Public Health. 2026 Aug 14;14:1883113. doi: 10.3389/fpubh.2026.1883113 (PMC13522205; doi:10.3389/fpubh.2026.1883113)
Supplement: Supplementary file 2 [file Supplementary_file_2.docx]

| **Health Literacy Scale** | | |
| --- | --- | --- |
| **Questionnaire** | **Domain** | **Item** |
| HLS-EU16 measure | Healthcare (HC) | Find information on the treatment of illnesses that concern you? |
|  |  | Find out where to get professional help when you are ill? |
|  |  | Understand what your doctor says to you? |
|  |  | Understand your doctor's or pharmacist's instructions on how to take a prescribed medicine? |
|  |  | Judge when you may need to get a second opinion from another doctor? |
|  |  | Use the information the doctor gives you to make decisions about your illness? |
|  |  | Follow instructions from your doctor or pharmacist? |
|  |  |  |
|  | Disease Prevention (DP) | Find information on how to manage mental health problems like stress or depression? |
|  |  | Understand health warnings about behavior such as smoking, low physical activity, and drinking too much? |
|  |  | Understand why you need health screenings? |
|  |  | Judge if the information provided on health risks in the media is reliable? |
|  |  | Decide how you can protect yourself from illness based on information in the media? |
|  |  |  |
|  | Health Promotion (HP) | Find out about activities that are good for your mental well-being? |
|  |  | Understand advice on health from family members or friends? |
|  |  | Understand information in the media on how to get healthier? |
|  |  | Judge which everyday behavior is related to your health? |
|  |  | Cronbach's alpha = 0.916 |
