# Supplementary material for: eHealth literacy mediates the relationship between health literacy and concussion attitudes among parents in South Korea
Source: Front Public Health. 2026 Aug 14;14:1883113. doi: 10.3389/fpubh.2026.1883113 (PMC13522205; doi:10.3389/fpubh.2026.1883113)
Supplement: Supplementary file 3 [file Supplementary_file_3.docx]

**EHEALS**

**We would like to ask about your experiences and opinions regarding using the Internet to obtain health-related information. For each question below, please select the response that best reflects your current thoughts and experiences.**

| Questionnaires | Strongly Disagree | Disagree | Undecided | Agree | Strongly agree |
| --- | --- | --- | --- | --- | --- |
| 1. I know what health resources are available on the Internet |  |  |  |  |  |
| 2. I know where to find helpful health resources on the Internet |  |  |  |  |  |
| 3. I know how to find helpful health resources on the Internet |  |  |  |  |  |
| 4. I know how to use the Internet to answer my questions about health |  |  |  |  |  |
| 5. I know how to use the health information I found on the Internet to help me |  |  |  |  |  |
| 6. I have the skills I need to evaluate the health resources I find on the Internet |  |  |  |  |  |
| 7. I can tell high quality health resources from low quality health resources on the Internet |  |  |  |  |  |
| 8. I feel confident in using information from the Internet to make health decisions |  |  |  |  |  |
