# Supplementary material for: eHealth literacy mediates the relationship between health literacy and concussion attitudes among parents in South Korea
Source: Front Public Health. 2026 Aug 14;14:1883113. doi: 10.3389/fpubh.2026.1883113 (PMC13522205; doi:10.3389/fpubh.2026.1883113)
Supplement: Supplementary file 4 [file Supplementary_file_4.docx]

| **Contents of knowledge, and attitudes toward concussions questionnaires** | | |
| --- | --- | --- |
| **Questionnaire** | **Domain** | **Item** |
| Concussion Knowledge | Signs or symptoms of concussions | Abnormal sense of smell (false) |
|  |  | Abnormal sense of taste (false) |
|  |  | Problems remembering things (true) |
|  |  | Joint stiffness (false) |
|  |  | Blurred vision (true) |
|  |  | Black eye (false) |
|  |  | Bleeding from the ear (false) |
|  |  | Bleeding from the mouth (false) |
|  |  | Bleeding from the nose (false) |
|  |  | Confusion (true) |
|  |  | Fever (false) |
|  |  | Dizziness (true) |
|  |  | Loss of consciousness (true) |
|  |  | Nausea (true) |
|  |  | Numbness or tingling of arms (true) |
|  |  | Skin rash (false) |
|  |  | Weakness in neck movements (false) |
|  |  | Chest pain (false) |
|  |  | Trouble understanding things (true) |
|  | General knowledge | A concussion only occurs if an athlete loses consciousness (false) |
|  |  | The brain is the body part hurt/injured with a concussion (true) |
|  |  | Consequences of returning to activity too soon |
|  |  | No complications exist (false) |
|  |  | One may be more likely to get another concussion (true) |
|  |  | Brain damage (true) |
|  |  | Skin rash (false) |
|  |  | Consequences of suffering multiple concussions |
|  |  | No complications exist (false) |
|  |  | One may be more likely to get another concussion (true) |
|  |  | Brain damage (true) |
|  |  | Skin rash (false) |
|  |  | Cronbach's alpha = 0.633 |
| Attitude towards concussions |  | How serious do you think it is when you experience a headache or dizziness after a blow to your head or body? |
|  |  | How important do you think it is to not participate in physical activity (game or practice) when experiencing signs and symptoms of concussions? |
|  |  | How important do you think it is to be informed about how concussions happen? |
|  |  | How important do you think it is to be informed about how concussions can be prevented? |
|  |  | How important do you think it is to know the steps to follow if an athlete has a concussion? |
|  |  | How important do you think it is to report possible signs and symptoms to a medical professional (e.g., doctor, athletic trainer) or coach? |
|  |  | Your level of agreement with the following statement: Athletes are undereducated about concussion |
|  |  | Cronbach's alpha = 0.87 |
